# Comparative study of magnesium, sodium valproate, and concurrent magnesium-sodium valproate therapy in the prevention of migraine headaches: a randomized controlled double-blind trial

**DOI:** 10.1186/s10194-021-01234-6

**Published:** 2021-04-07

**Authors:** Samira Khani, Seyed Amir Hejazi, Mehdi Yaghoubi, Ehsan Sharifipour

**Affiliations:** 1 Department of Neurology, Neurosciences Research Center (NSRC), Qom University of Medical Sciences, Shahid-Beheshti hospital, Shahid-Beheshti street, Qom, Iran; 2 Cellular and Molecular Research Center, Qom University of Medical Sciences, Qom, Iran

**Keywords:** Magnesium, Migraine, Prophylaxis, Sodium valproate

## Abstract

**Objective:**

This study aimed to assess the efficacy of concurrent magnesium-sodium valproate therapy and compare it with either magnesium or sodium valproate alone in migraine prophylaxis.

**Materials and methods:**

This randomized single-center double-blind parallel-group controlled clinical trial study was conducted on migraine patients within the age range of 18–65 years. The subjects with at least four monthly attacks were randomly assigned to group A (*n* = 82) sodium valproate, group B (*n* = 70) magnesium with sodium valproate, and group C (*n* = 70) magnesium. The patients passed a one-month baseline without prophylactic therapy and then received a 3-month treatment. The characteristics of migraine, including frequency, severity, duration of the attacks, and the number of painkillers taken per month, were monthly recorded in each visit. The Migraine Disability Assessment (MIDAS) and Headache Impact Test-6 (HIT-6) scores were recorded at the baseline and after 3 months of treatment in each group. Within- and between-group analyses were performed in this study.

**Results:**

The obtained results revealed a significant reduction in all migraine characteristics in all groups compared to those reported for the baseline (*P* <  0.001). Intragroup data analysis indicated that there was no statistically significant difference in headache frequency between groups A and B in the third month (*P* = 0.525); nevertheless, three other parameters showed a significant reduction in group B, compared to those reported for group A in the third month (*P* <  0.05). On the other hand, group C could not effectively reduce measured parameters in the patients, compared to groups A and B after 3 months (*P* <  0.001). Furthermore, the MIDAS and HIT-6 scores significantly diminished in groups A, B, and C compared to those reported at the baseline (*P* <  0.001), and these changes were more significant in groups A and B than in group C (*P* <  0.001).

**Conclusion:**

The obtained results of this study revealed that magnesium could enhance the antimigraine properties of sodium valproate in combination therapy and reduce the required valproate dose for migraine prophylaxis.

## Introduction

Migraine as a primary headache disorder with substantial pain is included in the 20 most disabling diseases according to the World Health Organization [39]. Epidemiologic studies have indicated that the prevalence of migraine is about 14% in Iran similar to or even higher than that reported worldwide [11]. Lifestyle management, acute treatment, and preventive treatment are three approaches to treat migraine [44]. Prophylactic therapy for migraine is recommended in patients with four or more attacks per month, eight or more headache days a month, debilitating headaches, and medication-overuse headaches [15].

A reduction in the frequency of headaches about 50% or more, reduced intensity, and improved response to symptomatic medication can be three outcomes of preventive therapy [28]. However, a lack of effectiveness, adverse effects, and poor compliance are also common in these patients [16, 35]. Beta-blockers, calcium-channel blockers, anticonvulsants, selective serotonin reuptake inhibitors, tricyclic antidepressants, and angiotensin blockers are some of the medications used for migraine prophylaxis. Furthermore, botulinum toxin, flunarizine, vitamins, minerals, and herbal agents were also suggested in many studies [14, 44].

Sodium valproate is one of the FDA-approved antiepileptic drugs (AEDs) for the prevention of migraine [27]. Several functions have been proposed for the antimigraine action of sodium valproate in such patients; nevertheless, the exact mechanism is not completely perceived due to its various biochemical effects and multifactorial nature of migraine pathophysiology [6, 37, 38]. Despite the proven efficacy of valproate in the prevention of migraine, poor compliance with therapy has been observed due to its side effects, such as fatigue, dizziness, nausea, tremor, and weight gain [2, 29].

Magnesium is a cofactor in more than 300 biochemical reactions and helps to maintain normal nerve and muscle functions [12]. In recent years, studies have focused on the clinical use of magnesium as a prophylactic regimen for migraine due to good efficacy and tolerability in patients [9]. Oral magnesium supplementation has been reported with level B evidence for its efficacy in the prophylactic therapy of episodic migraine based on the American Academy of Neurology Guidelines [8].

Although there has been existing evidence on the effectiveness of magnesium in migraine prophylaxis, no study has evaluated magnesium potency in increasing sodium valproate efficacy in combination therapy of migraine. The current study was the first attempt to compare the efficacy of combination therapy of magnesium and sodium valproate with each treatment alone in migraine prophylaxis.

## Methods

### Study design

This single-center placebo-controlled double-blind randomized trial study (registered in Iranian Registry of Clinical Trials with registry no. IRCT2015081923685N1) was conducted on migraine patients on October 11 in 2015. Ethical approval for this study was obtained from the Ethics Committee of Qom University of Medical Sciences in Qom, Iran (special approval ID: IR.MUQ.REC.1394.73) on August 26 in 2015 in accordance with the Helsinki Declaration. This study was conducted at the Neurological Special Clinic of Shahid Beheshti Hospital in Qom. The research assistant evaluated eligibility obtained informed consent and enrolled the participants.

A total of 260 patients entered into the present study with signed consent about the inclusion and exclusion criteria. The patients were recruited from those referred to an outpatient neurological clinic and also through advertisements on social networks (i.e., Telegram and WhatsApp). The patients were randomly assigned into three groups, including the intervention groups A, B, and C. Intervention group A received a 200 mg sodium valproate tablet twice a day orally for 12 weeks and a placebo tablet twice a day orally for 12 weeks. Intervention group B received a 200 mg sodium valproate tablet twice a day orally for 12 weeks and a 250 mg magnesium oxide tablet twice a day orally for 12 weeks. Intervention group C received a 250 mg magnesium oxide tablet twice a day orally for 12 weeks and a placebo tablet twice a day orally for 12 weeks.

All the patients underwent a 1-month baseline period (without prophylactic medication) to assess the frequency of attacks, severity of attacks, and amount of collaboration with the physician. The study duration since the onset of drug prescription would be 3 months, and the patients were monthly followed. The concurrent administration of acute abortive treatment was not prohibited during this study; however, the patients should record the dose of pain killer in diaries. Headache diaries and headache questionnaires (i.e., the Migraine Disability Assessment [MIDAS] and Headache Impact Test-6 [HIT]) were used to obtain clinical information. The paper diary which was used to record information about the attacks of migraine during the baseline and treatment period, included the Wong-Baker Faces Pain Rating Scale for severity assessment and data about the frequency, duration, days with migraine per month, and number of painkillers used per month. The patients should daily complete the diary, and the research assistant called the patients every week to ensure patient compliance.

#### Primary efficacy measures

The three groups were evaluated to compare the efficacy of the three treatment schedules on migraine frequency at the end of the baseline, and in the first, second, and third months after the treatment.

#### Secondary efficacy measures

Migraine severity, duration of attacks and number of painkillers used per month were compared among the three treatment groups. Also, the changes in the MIDAS and HIT-6 scores since the baseline were calculated after a 3-month treatment in each group. The HIT-6 was used to assess the severity of headache impact on the patient life, and the HIT-6 score is within the range of 36–78 [34]. The MIDAS questionnaire was also utilized to determine migraine-related disabilities over 3 months. The MIDAS scores include several ranges, namely 0–5 (MIDAS grade I: minor or no disability), 6–10 (MIDAS grade II: mild disability), 11–20 (MIDAS grade III: moderate disability), and 21 or higher (MIDAS grade IV: severe disability) [3, 18].

### Inclusion and exclusion criteria

The inclusion criteria were the diagnosis of migraine according to the latest International Headache Society criteria, history of migraine with or without aura for at least 6 months, age range of 18–65 years, and experience of at least four monthly attacks. The exclusion criteria were non-migraine headaches, total number of headache days per month higher than 15, overuse of analgesics in migraine attacks (i.e., the use of ergots, nonsteroidal anti-inflammatory drugs, and triptans higher than 8 days a month), substance and alcohol dependence, illiteracy of patients and their family (unable to fill diaries), pregnancy and nursing, history of magnesium or sodium valproate intolerance, history of renal, liver, and chronic diseases, elevated liver enzymes in the first sampling more than two times the normal, neurologic disorders other than migraine, having other comorbidities i.e. fibromyalgia or etc., use of supplements containing magnesium, use of herbal antimigraine, and use of antidepressant and antipsychotic medications.

### Randomization and blinding

Block randomization sampling was used in the present study. Each subject received an ID code, and the medications were delivered to the patients based on the order of the blocks in completely identical containers without the labels, marked as A, B, or C, to complete the sample size. The clinicians and patients were blinded to the reception of the drugs during the study period.

### Sample size and statistical methods

The required sample size for the study was calculated at least 67 subjects in each group with a power of 80% and the significance level of 5%; nevertheless, the recovery rates in the two groups of the study were 65% and 38.1%, based on the previous trials, respectively [22]. Therefore, 260 patients were divided into three groups via random divisions (A = 88, B = 86, C = 86) considering the dropout rate.

All the statistical analyses were performed using SPSS software (version 20). Firstly, the normal distribution of the data at the beginning of the study was investigated using the Kolmogorov-Smirnov test. Descriptive characteristics, such as mean and standard deviation, were used to explain the statistical results. Patient characteristics (e.g., gender, migraine type, and family history) were analyzed using the Pearson’s Chi-square test. Intergroup comparisons were performed using a paired student’s t-test. The analysis of variance and Tukey’s post-hoc test were used to compare the values among the three treatment groups. A *p*-value of less than 0.05 was considered statistically significant.

## Results

**Fig. 1:**
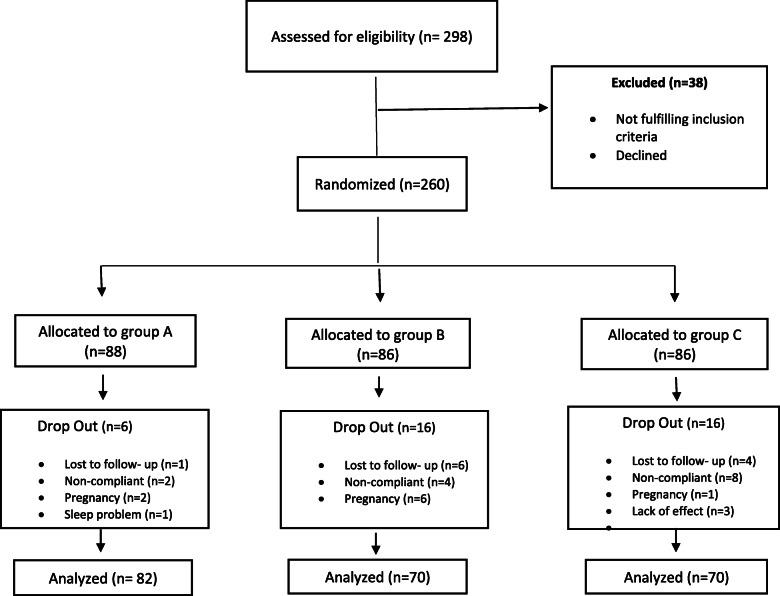
CONSORT flow diagram of participants through the study

A total of 298 patients with recurrent migraine were enrolled in the present study. After careful screening, 260 patients were randomized to different treatment groups (i.e., A, B, and C). Finally, 222 patients (including 125 females and 97 males) completed the study. The consort diagram of the study is presented in Fig. 1. As shown in Table 1, there was no significant difference in demographics, migraine type, and headache history at the baseline in various treatment groups.

**Table 1:** Patient Demographic characteristics at baseline

Characteristics		A (*n* = 82)	B (*n* = 70)	C (n = 70)	*p*-value
Sex (Female), n		41	42	42	0.35
Age, y		35.16 ± 8.21	37.11 ± 6.56	34.41 ± 6.19	0.27
BMI (Kg/m^2^)		24.78 ± 8.34	24.76 ± 8.54	24.42 ± 7.70	0.17
Migraine type, n	Without Aura	51	39	44	0.62
	With Aura	31	31	26	
Headache history, y		5.23 ± 2.49	5.73 ± 2.14	4.89 ± 3.14	0.25
Family history of migraine, n	Yes	55	43	47	0.71
	No	27	27	23	

**Fig. 2:**
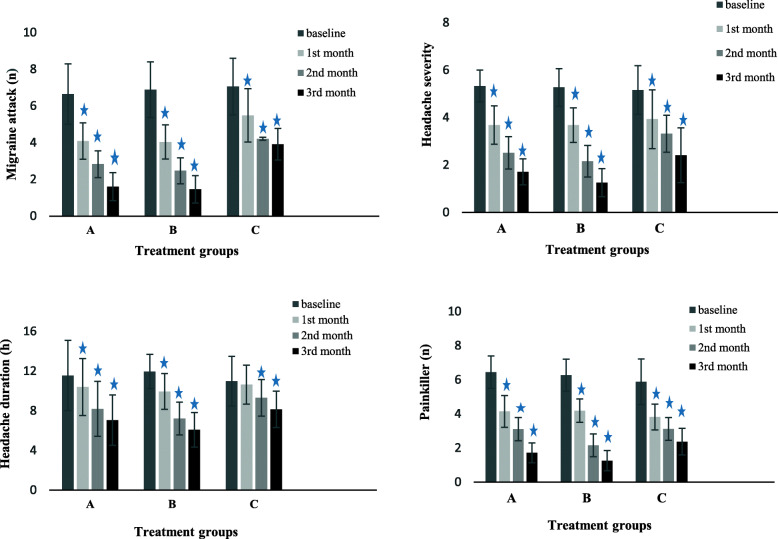
Migraine characteristics of the three groups in different treatment periods; *P values≤0.05 were considered statistically significant vs baseline [measured using sample (paired) t test]. A: Sodium Valproate; B: Sodium Valproate +Magnesium; C: Magnesium

**Fig. 3:**
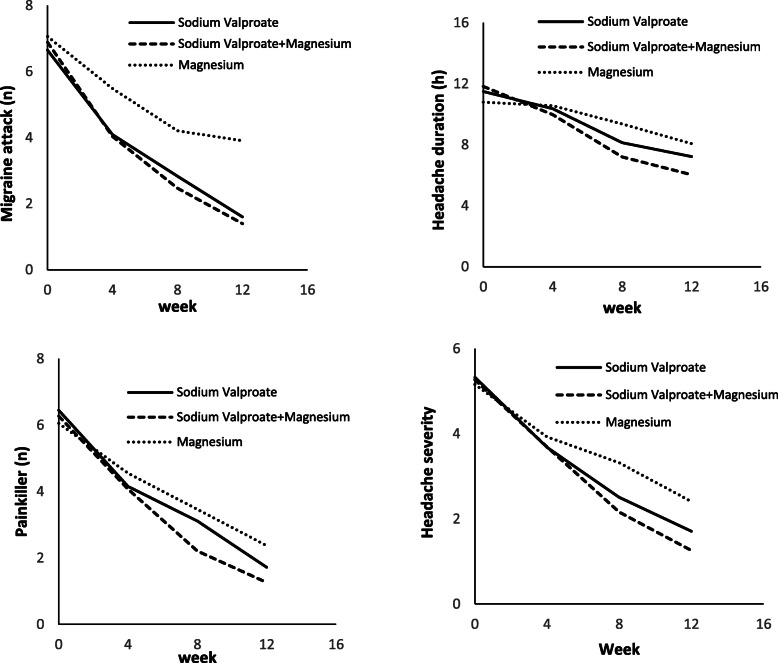
Intra group comparison of duration, severity, number of attack and painkiller in different treatment periods

The patients were evaluated in terms of headache frequency, headache severity, headache duration, and number of painkillers taken per month after the onset of the treatment for 3 months. The intergroup and intragroup analyses of the data were performed. The obtained results showed a significant reduction in the measured parameters in the three arms of the study after 3-month of treatment, compared to those reported for the baseline (*P* <  0.001). However, a significant reduction in headache duration in group C was not demonstrated during the first month of the study (*P* = 0.153) (Figs. 2 and 3).

Intragroup data analysis indicated that there was no statistically significant difference in headache frequency between groups A and B in the first (*P* = 0.972) and third (*P* = 0.525) months; nonetheless, group B revealed a significant reduction in the second month, compared to group A (*P* = 0.029). Furthermore, group C indicated a significant difference in headache frequency in comparison to groups A and B over 3 months (*P* <  0.001) (Table 2).

**Table 2:** Intragroup monthly basis analysis of Migraine attack, Headache severity, Headache duration and Painkiller number (A: Sodium Valproate; B: Sodium Valproate +Magnesium; C: Magnesium); *P values≤0.05 were considered statistically significant

Group	A	B	C	A vs B		A vs C				B vs C		
				*p*- value	Mean Difference	95% CI	*p*- value	Mean Difference	95% CI	*p*- value	Mean Difference	95% CI
Migraine attack (n), mean ± SD												
baseline	6.65 ± 1.65	6.89 ± 1.52	7.06 ± 1.54	0.621	− 0.24	− 0.85 to 0.37	0.248	− 0.41	−1.02 to 0.20	0.797	− 0.17	− 0.80 to 0.46
After 1 month	4.09 ± 0.99	4.04 ± 0.93	5.49 ± 1.45	0.972	0.05	− 0.40 to 0.48	< 0.001	− 1.40	− 1.84 to − 0.96	< 0.001	−1.45	− 1.90 to 0.99
After 2 month	2.83 ± 0.73	2.47 ± 0.71	4.21 ± 0.08	0.029	0.36	0.03 to 0.69	< 0.001	− 1.38	− 1.71 to − 1.06	< 0.001	− 1.74	−2.08 to − 1.40
After 3 month	1.60 ± 0.76	1.40 ± 0.75	3.91 ± 0.86	0.525	0.20	− 0.17 to 0.45	< 0.001	−2.31	− 2.62 to − 2.01	< 0.001	−2.51	− 2.77 to − 2.14
Headache severity, mean ± SD												
baseline	5.33 ± 0.67	5.27 ± 0.79	5.16 ± 1.02	0.885	0.06	− 0.25 to 0.38	0.424	0.17	− 0.15 to 0.49	0.732	0.11	− 0.22 to 0.43
After 1 month	3.68 ± 0.81	3.69 ± 0.73	3.93 ± 1.24	0.999	− 0.01	−0.37 to 0.35	0.243	−0.25	− 0.61 to 0.11	0.287	− 0.24	−0.62 to 0.13
After 2 month	2.51 ± 0.68	2.16 ± 0.67	3.32 ± 0.78	0.01	0.35	− 0.06 to 0.61	< 0.001	− 0.81	− 1.08 to − 0.54	< 0.001	− 1.16	− 1.44 to 0.87
After 3 month	1.71 ± 0.55	1.26 ± 0.59	2.41 ± 1.15	0.002	0.45	− 0.13 to 0.75	< 0.001	−0.70	− 1.00 to − 0.39	< 0.001	− 1.15	− 1.46 to − 0.82
Headache duration (h), mean ± SD												
baseline	11.56 ± 3.53	11.96 ± 1.73	10.99 ± 2.49	0.649	− 0.40	− 1.45 to 0.66	0.403	0.57	− 0.48 to 1.63	0.093	0.97	−0.12 to 2.07
After 1 month	10.39 ± 2.87	9.95 ± 1.80	10.64 ± 1.96	0.481	0.44	− 0.45 to 1.31	0.779	− 0.25	− 1.13 to 0.63	0.185	− 0.69	− 1.60 to 0.23
After 2 month	8.19 ± 2.76	7.22 ± 1.66	9.30 ± 1.84	0.019	0.97	0.12 to 1.80	0.006	−1.11	− 1.94 to − 0.26	< 0.001	− 2.08	− 2.94 to − 1.20
After 3 month	7.06 ± 2.53	6.08 ± 1.75	8.15 ± 1.83	0.013	0.98	0.17 to 1.77	0.004	− 1.09	− 1.90 to − 0.29	< 0.001	−2.07	− 2.90 to − 1.23
Painkiller (n),mean ± SD												
baseline	6.45 ± 0.95	6.27 ± 0.94	6.05 ± 1.48	0.601	0.18	−0.26 to 0.62	0.083	0.40	−0.04 to 0.84	0.489	0.22	−0.24 to 0.68
After 1 month	4.15 ± 0.93	4.07 ± 0.73	4.55 ± 1.13	0.871	0.08	− 0.29 to 0.44	0.031	− 0.40	− 0.76 to − 0.03	0.010	−0.48	− 0.85 to − 0.09
After 2 month	3.11 ± 0.68	2.21 ± 0.75	3.46 ± 0.89	< 0.001	0.90	0.60 to 1.19	0.017	− 0.35	−0.64 to − 0.05	< 0.001	− 1.25	− 1.55 to − 0.93
After 3 month	1.72 ± 0.58	1.26 ± 0.59	2.37 ± 0.78	< 0.001	0.46	0.20 to 0.71	< 0.001	−0.65	− 0.89 to − 0.39	< 0.001	− 1.11	−1.36 to − 0.84

No significant difference in headache severity was observed in the first month among the three groups; nevertheless, severity reduction was significant in group B in comparison to those reported for group A (*P* = 0.01 and *P* = 0.002) and group C (*P* <  0.001) in the second and third month, respectively. Severity reduction was also higher in group A, compared to that reported for group C in the second and third months (*P* <  0.001) (Table 2).

The three groups had no significant difference in headache duration in the first month; however, groups A and B were both significantly better than group C regarding headache duration with *p*-values of 0.006 and less than 0.001 in the second month and *p*-values of 0.004 and less than 0.001 in the third month, respectively. In addition, headache duration significantly differed between groups A and B in the second (*P* = 0.019) and third (*P* = 0.013) months (Table 2).

The number of painkillers had no statistically significant difference between groups A and B in the first month (*P* = 0.871); however, it was significantly lower in groups A (*P* = 0.031) and B (*P* = 0.010), compared to that reported for group C in the first month. The number of painkillers was significantly lower in group B in comparison to those reported for group A in the second and third months (*P* <  0.001). Both groups A and B used significantly lower numbers of painkillers than group C in the second (*P* = 0.017 and *P* <  0.001, respectively) and third (*P* <  0.001) months (Table 2).

**Fig. 4:**
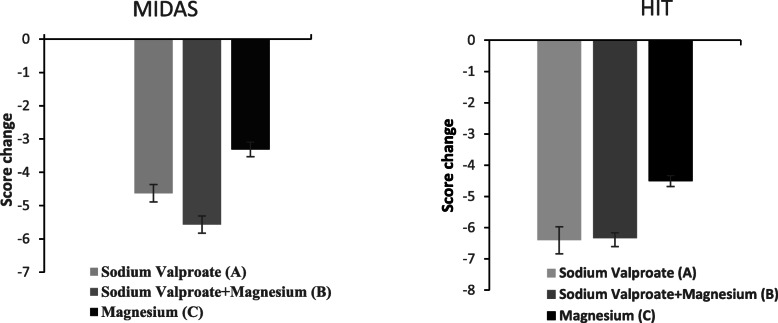
Comparison of MIDAS and HIT-6 score change between three treatment groups. MIDAS change, P value: (A Vs B = 0.023, A Vs C = 0.001, B Vs C < 0.001). HIT change, P value: (A Vs B = 0.999, A Vs C < 0.001, B Vs C < 0.001)

The MIDAS and HIT-6 scores (Table 3) were significantly lower in all the treatment groups in comparison to those measured at the baseline (*P* < 0.001). The intragroup analysis of MIDAS and HIT-6 changes also indicated that groups A and B had significantly higher changes in scores than group C (*P* < 0.001). However, no significant difference was observed between groups A and B in HIT score change (*P* = 0.999). The changes in MIDAS scores were significantly different between the two groups (group A vs. group B; *P* = 0.023) (Fig. 4).

**Table 3:** Analysis of MIDAS and HIT score in a different group (A: Sodium Valproate; B: Sodium Valproate +Magnesium; C: Magnesium) compared to baseline

	A		*P*-value	B		*P*-value	C		*P*-value
	Pre-intervention	Post-intervention		Pre-intervention	Post-intervention		Pre-intervention	Post-intervention	
MIDAS	21.74 ± 4.44	17.11 ± 4.06	< 0.001	21.68 ± 3.72	16.11 ± 3.87	< 0.001	22.13 ± 1.88	18.81 ± 1.76	< 0.001
HIT-6	56.72 ± 4.59	49.91 ± 4.58	< 0.001	56.89 ± 3.84	50.50 ± 3.27	< 0.001	57.54 ± 2.13	53.03 ± 1.88	< 0.001

*MIDAS* migraine disability assessment score; *HIT-6* Headache Impact Test-6 score

## Discussion

The current study compared the efficacy of sodium valproate (group A), sodium valproate plus magnesium oxide (group B), and magnesium oxide (group C) in the migraine prophylaxis of patients within the age range of 18–65 years. Furthermore, the MIDAS and HIT6 scores were compared among the three treatment groups. The obtained results showed that the combination of magnesium and sodium valproate had an appropriate efficacy in migraine prophylaxis, as headache severity, duration of headache, and amount of utilized painkillers were significantly lower in group B, compared to those reported for group A. Moreover, the MIDAS scores further reduced in group B than those reported for group A, and the changes in HIT scores were not significantly different between groups A and B. Furthermore, by the addition of magnesium to valproate (group B), the sodium valproate dose significantly didn’t increase, compared to that reported for group A.

Sodium valproate belongs to the class of AEDs with an important role in the treatment of migraine. Increasing GABA activity and inhibiting NMDA-evoked neuroexcitatory signals are two main mechanisms of valproate in blocking cortical spreading depression during a migraine attack. It can inhibit GABA-degrading enzymes (i.e., aminotransferase and succinic semialdehyde) and increase the neuro-inhibitory activity of GABA. In addition, the active metabolites of valproate can activate the GABA-synthesizing enzyme (i.e., glutamic acid decarboxylase) and further increase GABA activity over time. Furthermore, valproate decreases neurogenic inflammation by debilitating plasma extravasation of vasoactive neuropeptides, such as substance P, CGRP, and neurokinin A [27, 43].

There is abundant evidence that valproate is effective in the prevention of migraine attacks; however, different response rates were reported in various studies [26, 33]. Ichikawa et al. demonstrated that different factors, such as the history of hyperlipidemia, allergy, and psychiatric disorders, are involved in the clinical responses to valproate [20]. Sodium valproate has been used in doses within the range of 500–1000 mg/day in migraine prevention trials [27]. Nevertheless, in the current study, a lower dose of 200 mg was prescribed twice a day for patients with an acceptable response. Other studies conducted on the Iranian population also revealed that the therapeutic effect is achieved in migraine prophylaxis using a daily dose of sodium valproate within the range of 200–500 mg [4, 16, 19].

The effectiveness of magnesium in migraine prophylaxis was firstly investigated by [10] [10]. Other trials also reported different results of magnesium efficacy in migraine patients [23, 40]. Slavin et al. demonstrated that the inadequate consumption of magnesium intake is associated with migraine in American adults within the age range of 20–50 years [36]. There is evidence that the concentrations of magnesium in the occipital lobes of patients with migraine and cluster headaches are lower than those reported for healthy controls [25].

Therefore, further clinical trials were suggested to clarify the exact efficacy of magnesium in the prevention of migraine. It is proposed that magnesium is linked to migraine pathogenesis by counteracting both vascular and neurogenic mechanisms of migraine [41]. Magnesium may be effective in migraine through the regulation of neuronal excitability because magnesium not only acts as a physiologic calcium-antagonist but also inhibits NMDA receptors and glutamate-dependent excitatory pathways [7, 17].

In addition, magnesium can regulate neurotransmitter release and substance P release and reduce free radical accumulation within the cell and vasoconstriction [1, 10, 45]. Magnesium through modulating mitochondrial oxidative phosphorylation, 5-HT neurotransmission, and NO system, regulating the uptake of glutamate into astrocytes, and blocking of NMDA receptor can be effective in migraine-preventive therapy [38].

Magnesium has numerous effects on the nervous system such as analgesic effects in neuropathic pain [30] and visceral inflammatory pain [42]. Magnesium can decrease inflammation by inhibiting pro-inflammatory intracellular signaling, such as the nuclear factor kappa B pathway [24]. Other mechanisms, including the inhibition of voltage-gated calcium channels, connexin channels, and other ion channels, can be involved in the prevention of migraine [17]. Based on the evidence, magnesium was strongly recommended by the Canadian Headache Society in migraine prophylaxis. The Swiss Headache Society also suggests magnesium to children and pregnant women with migraine [28].

In a crossover study conducted by Karimi et al., the comparison of efficacy between magnesium oxide and sodium valproate was carried out for migraine prophylaxis. The results showed that 500 mg/day magnesium has comparable efficacy to 400 mg/day valproate in migraine prophylaxis [21]. However, the obtained results of the present study indicated that valproate was significantly more effective than magnesium in the reduction of migraine frequency and severity, duration of attacks, painkiller number, and MIDAS and HIT scores.

According to the literature, it was shown that the addition of magnesium to valproic acid in magnesium valproate can efficiently reduce calcium ion conductance, activate the Na^+^/K^+^ ion pump, and modulate the NMDA receptors of the neuronal membrane in epilepsy, compared to valproate alone [5]. Animal studies also demonstrated that magnesium enhances the anticonvulsant potential of a subprotective dose of valproic acid in pentylenetetrazol-treated rats by the improvement of redox balance and modulation of some brain excitatory amino acids, such as aspartate, asparagine, and glycine [31].

It should be noted that epilepsy and migraine have some similar pathophysiological mechanisms, including the imbalance between GABA-mediated inhibition, excitatory glutamate-mediated transmission, and abnormal function of voltage-gated sodium and calcium channels [32]. The results of in vitro studies demonstrated that magnesium improves valproic acid efficacy against 4-aminopyridine-induced ictal activity [13]. The findings of the present clinical study also confirmed this idea and indicated that a combination of magnesium and low-dose valproate (200 mg) have appropriate efficacy in migraine prophylaxis without any reported exacerbation of side effects.

### Study limitations

Some participants in this study did not contribute to blood sampling. A lack of complete data about serum magnesium levels limits the analysis of the correlation between the serum levels of magnesium and efficacy of treatment in the three groups. Furthermore, the assessment of adverse effects during the study was not completely carried out due to faulty reports; therefore, the precise analysis was impossible.

## Conclusion

The results of the present study revealed that combination treatment with a low dose of sodium valproate and magnesium has desired efficacy in the reduction of migraine frequency, severity, duration of attacks, and number of utilized painkillers. Considering these promising results, it also indicated that by the addition of magnesium to valproate, sodium valproate unchanged low dose improves its efficacy. Consequently, the development of a new combination drug (Magnesium Valproate) in this manner, can be a more potent and safe therapy for migraine prophylaxis.

## Data Availability

The supporting data is available.
